# Investigation of the Complexes Formed between PARP1 Inhibitors and PARP1 G-Quadruplex at the Gene Promoter Region

**DOI:** 10.3390/ijms22168737

**Published:** 2021-08-14

**Authors:** Sabrina Dallavalle, Salvatore Princiotto, Luce M. Mattio, Roberto Artali, Loana Musso, Anna Aviñó, Ramon Eritja, Claudio Pisano, Raimundo Gargallo, Stefania Mazzini

**Affiliations:** 1 Department of Food, Environmental and Nutritional Sciences (DEFENS), University of Milan (Università degli Studi di Milano), 20133 Milan, Italy; sabrina.dallavalle@unimi.it (S.D.); salvatore.princiotto@unimi.it (S.P.); luce.mattio@unimi.it (L.M.M.); loana.musso@unimi.it (L.M.); 2 National Institute of Fundamental Studies, Kandy 20000, Sri Lanka; 3 Scientia Advice di Roberto Artali, 20832 Desio, Italy; roberto.artali@scientia-advice.com; 4 Institute for Advanced Chemistry of Catalonia (IQAC), CSIC, Networking Center on Bioengineering, Biomaterials and Nanomedicine (CIBER-BBN), 08034 Barcelona, Spain; aaagma@cid.csic.es (A.A.); recgma@cid.csic.es (R.E.); 5 Biogem, Research Institute, Ariano Irpino, 83100 Avellino, Italy; claudio.pisano@biogem.it; 6 Department of Chemical Engineering and Analytical Chemistry, University of Barcelona, 08028 Barcelona, Spain; raimon_gargallo@ub.edu

**Keywords:** NMR spectroscopy, CD, G-quadruplex, molecular modeling, PARP1 inhibitors, fluorescence titration, PARP1 promoter, dual-targeting

## Abstract

DNA repair inhibitors are one of the latest additions to cancer chemotherapy. In general, chemotherapy produces DNA damage but tumoral cells may become resistant if enzymes involved in DNA repair are overexpressed and are able to reverse DNA damage. One of the most successful drugs based on modulating DNA repair are the poly(ADP-ribose) polymerase 1 (PARP1) inhibitors. Several PARP1 inhibitors have been recently developed and approved for clinical treatments. We envisaged that PARP inhibition could be potentiated by simultaneously modulating the expression of PARP 1 and the enzyme activity, by a two-pronged strategy. A noncanonical G-quadruplex-forming sequence within the PARP1 promoter has been recently identified. In this study, we explored the potential binding of clinically approved PARP1 inhibitors to the G-quadruplex structure found at the gene promoter region. The results obtained by NMR, CD, and fluorescence titration confirmed by molecular modeling demonstrated that two out the four PARP1 inhibitors studied are capable of forming defined complexes with the PARP1 G-quadruplex. These results open the possibility of exploring the development of better G-quadruplex binders that, in turn, may also inhibit the enzyme.

## 1. Introduction

Poly (ADP-ribose) polymerase-1 (PARP1) is a nuclear enzyme involved in DNA repair processes [1,2]. The intervention of PARP1 takes place early in the steps of the DNA repair process, after activation by DNA nicks [3]. Upon poly-ADP ribosylation, the enzyme repairs break in single-strand DNA through a base excision repair pathway. Additionally, PARP1 is implicated in other cellular processes such as transcriptional regulation, chromatin remodeling, cell signaling and cell death [4,5].

PARP1 inhibition causes the so-called “synthetic lethality” in tumor cells with defective homologous recombination pathways and sensitizes tumor cells to DNA damaging chemotherapies, including multiple chemotherapy or radiotherapy approaches, which remain the backbone of treatment for most cancer patients [6]. Consequently, PARP1 has emerged as an attractive target for cancer therapy. By inhibiting the PARP-mediated repair of DNA lesions created by chemo- or radiotherapy, greater potency might be achieved.

**Figure 1:**
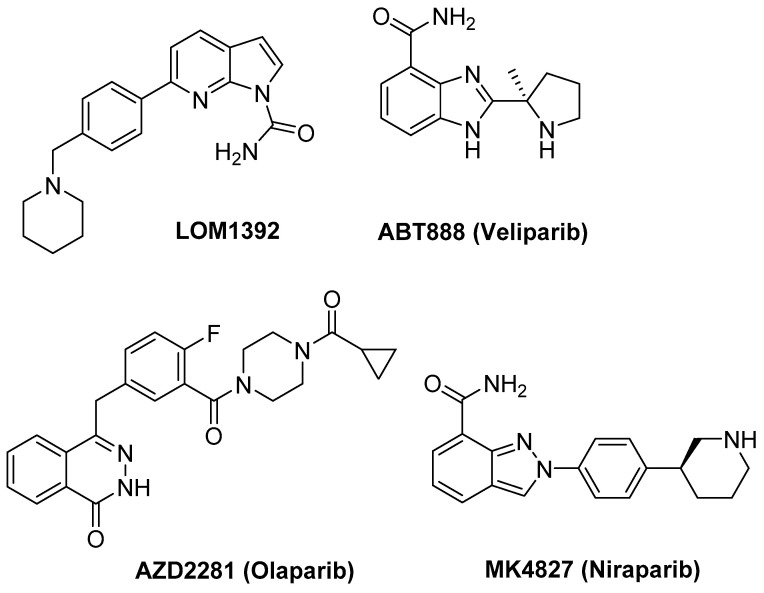
Chemical structures of the PARP1 inhibitors selected for the present study.

Several PARP1 inhibitors have been successfully developed, with Olaparib being the first one to be approved clinically for treating BRCA1/2-mutated cancers (Figure 1). The approval of Olaparib for ovarian cancer was followed by promising clinical results with other PARP inhibitors, including Veliparib, Rucaparib, and Niraparib. The last two and Talazoparib have also been approved and are in clinical use for the treatment of ovarian cancer. Some others are in advanced clinical trials as single agents or in combination with DNA-damaging drugs [7]. From a clinical point of view, Veliparib is the less active compound, followed by Olaparib and Niraparib with increased inhibitory activity [8].

Moreover, some of us are involved in the development of novel PARP1 inhibitors (Figure 1), one of the most active compounds being LOM1392 [9].

However, the emergence of resistance to PARP1 inhibitors, mediated by multiple molecular mechanisms, including changes in the expression of PARP1 itself [8], has generated the need for alternative strategies to selectively interfere with PARP1 activity.

An unexplored approach could be the identification of small molecules acting as transcriptional repressors of PARP1. The transcriptional repression of PARP1 could help in the prevention of diseases that occur due to the overexpression of PARP1 and subsequently influence the expression of other oncogenes.

In this context, recent findings have suggested a potential for interfering with PARP1 regulation via G-quadruplex targeting.

G-quadruplexes are four-stranded structures formed by G-rich nucleic acids comprising a stack of multiple guanine(G)-tetrads. A G-tetrad is a square planar arrangement of four guanines stabilized by Hoogsteen hydrogen bonds [10].

G4s are nonrandomly distributed through the genome, mainly being clustered in key regulatory sites such as gene promoters, gene bodies, and untranslated regions of highly transcribed genes, particularly those related to cancer [11]. In these genomic locations, G4s are linked to fundamental biological processes such as transcription, replication, genomic instability, and telomeres maintenance. A wide variety of G-quadruplex structures have been elucidated, differing in terms of strand orientations, glycosylic conformations, groove sizes, connecting loops, and number of tetrads. These structures have been extensively associated with cancer, playing an important role in telomere maintenance and the control of genetic expression of several oncogenes and tumor suppressors [11].

A link between the ligand-mediated stabilization of G-quadruplexes in gene promoters and transcriptional regulation has been proposed for several oncogenes. Therefore, quadruplex structures are considered attractive molecular targets for cancer therapeutics with novel mechanisms of action.

Recently, Chambers et al. identified noncanonical G-quadruplex-forming sequences in the promoter region of the PARP1 gene by genome-mapping experiments [12].

Successively, Sengar and coworkers [13] investigated the G-quadruplex structure formed by a 23-nucleotide G-rich sequence (TGGGGGCCGAGGCGGGGCTTGGG (termed as *TP3*)) located 125 nucleotides (nts) upstream of the transcription start site (TSS) in the PARP1 promoter by NMR spectroscopy. The study revealed a three-layered intramolecular (3 + 1) hybrid G-quadruplex folding topology, in which three strands are oriented in one direction and the fourth in the opposite direction. It should be noted that this structure exhibits unique structural features, such as an adenine bulge and a GGT base triple capping structure formed between the central edgewise loop, propeller loop, and 5′ flanking terminal.

The identification of the structural features of the PARP1 promoter offers a new and attractive opportunity to explore the therapeutic potential of PARP1 inhibition via G-quadruplex DNA targeting. The molecular features of the quadruplex could be exploited as distinct recognition elements to target this G-quadruplex structure with specificity. To the best of our knowledge, there are no examples of small molecules targeting the G-quadruplex structures in the PARP 1 promoter.

Given the uncharacterized role (inhibitory or stimulatory) of a TP3 G4 element in the PARP1 promoter, we hypothesized that it could be a target for the reduction of PARP1 expression. We envisaged that PARP inhibition could be potentiated by simultaneously modulating PARP 1 expression and the enzyme activity, by a two-pronged strategy. With this purpose, we investigated a small collection of current PARP inhibitors, which are known to possess a strong inhibitory activity toward the enzyme, in order to ascertain whether any of them were also able to stabilize the G4 structure of the promoter.

Herein, we reported an NMR, fluorescence, CD, and molecular modeling study focused on the interaction of selected small molecules with a noncanonical G4 located at the PARP1 promoter, 125 nucleotides upstream of the transcription start site (TSS) [13].

## 2. Results and Discussion

To study the association mode of ligands with G quadruplex structures located at the PARP1 promoter, we selected the clinically used drugs veliparip (ABT 888), olaparib (AZD 2281), and niraparib (MK4827). We also tested compound LOM1392, which belongs to a new class of PARP1 inhibitors with a 7-azaindole-1-carboxamide core. We have recently found that the compound potently inhibits the enzyme PARP1 (IC_50_ = 0.07 mM) [9] and, additionally, is a strong G-quadruplex binder [14].

### 2.1. ^1^H NMR Experiments and Molecular Modeling on the LOM1392, ABT888, MK4827, and AZD2281 Complexes with TP3-T6 (5′-D TGGGGT6CCGAGGCGGGGCTTGGG-3′)

We considered for our study the oligonucleotide (TP3-T6) that contains a G-to-T substitution at position 6 and shows a high-quality NMR spectrum very similar to that of TP3, indicating the formation of the same G-quadruplex-folded structure, having, however, an improved temporal stability upon exposure to room temperature [13]. The interaction of the known PARP-1 inhibitors LOM1392, ABT888, MK4827, and AZD2281 with TP3-T6 was studied at 20 mM of KH_2_PO_4_ and 70 mM of KCl (pH = 7.0).

^1^H NMR titration experiments were performed by adding increasing amounts of ligands to the TP3-T6 solution, with ratios R = [ligand]/[DNA] ranging from 0 to 2.0. In particular, we observed the ^1^H imino protons, which lie in a characteristic region between 10.5 and 12.0 ppm, and their perturbation during the titration experiments. TP3-T6 shows twelve well-resolved imino proton signals in the ^1^H NMR spectrum, consistent with the formation of a G-quadruplex with three G-quartets.

**Figure 2:**
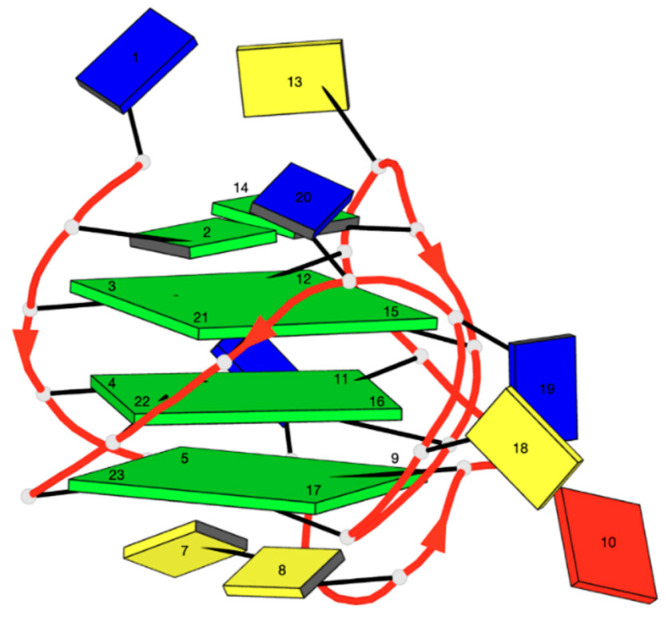
Schematic representation of TP3-T6 oligomer G-quadruplex.

**Figure 3:**
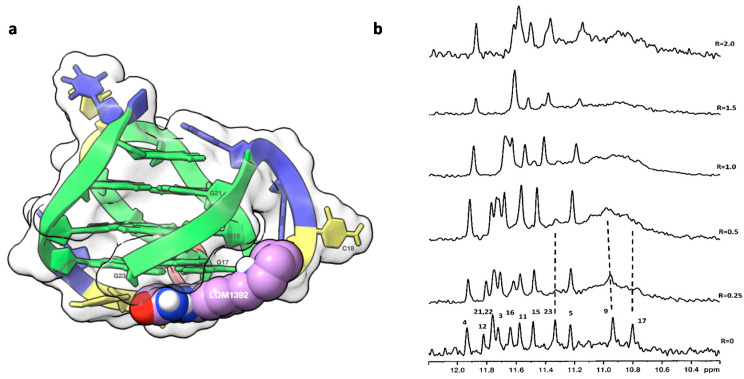
(**a**) Graphical representations of the complex and (**b**) imino proton region of the 1D NMR with LOM1392. The complex at TP3-T6 was obtained by molecular docking and optimized by molecular dynamics (MD), and it is represented as a side view of the ghostly-white solvent-accessible surface (SAS) of the TP3-T6 target. The nucleotides rendered in Scheme 1392 is represented as van der Waals (vdW) spheres. Drawing was created by using the Chimera-X software [15]. The imino proton regions of the 1D NMR titration spectra were recorded at 25 °C in H_2_O/D_2_O (9:1), 20 mM of KH_2_PO_4_, and 70 mM of KCl pH = 7.0, at different R = [LOM1392]/[DNA] ratios.

**Figure 4:**
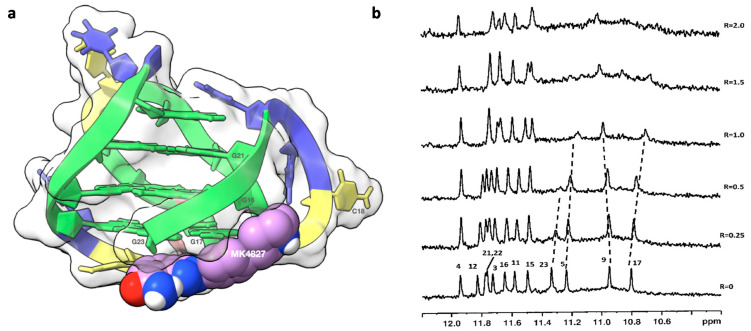
(**a**) Graphical representations of the complex and (**b**) imino proton region of the 1D NMR with MK4827. The complex at TP3-T6 was obtained by molecular docking and optimized by molecular dynamics (MD), and it is represented as a side view of the ghostly-white solvent-accessible surface (SAS) of the TP3-T6 target. Figure created by using the ChimeraX software [15]. The nucleotides are rendered in stick and filled rings: cytosine in yellow, guanine in green, and thymine in blue. MK4827 is represented as van der Waals (vdW) spheres. The imino proton regions of the 1D NMR titration spectra were recorded at 25 °C in H_2_O/D_2_O (9:1), 20 mM of KH_2_PO_4_, and 70 mM of KCl pH = 7.0, at different R = [MK4827]/[DNA] ratios.

The binding of LOM1392 and MK4827 to TP3-T6 (Figure 2) was evident from the line broadening of imino ^1^H NMR signals even at ratios of 0.25–0.5. A more pronounced effect was detected for G23 (11.30 ppm), G9 (10.91 ppm), and G17 (10.80 ppm) imino protons that belong to the 3′-end tetrad (Figure 3b and Figure 4b). At these low ratios, the remaining guanine residues, belonging to the middle and 5′-end tetrads, showed only a moderate change both in intensity and in chemical shifts. This is consistent with a selective recognition of the 3′-end.

Even at higher R ratios (R ≥ 1.0), no evidence of additional binding sites was detected. At R = 2.0, only a small number of imino proton signals were observed, in comparison with the 12 signals expected for a single species of a folded G-quadruplex. All these findings suggest that the ligands bind preferentially at the 3′-end, partially disrupting the G-quadruplex tetrad network (Figure 3b and Figure 4b). The subsequent loss of stability can be compensated by favorable interactions with the ligands. The broadening of the NMR signals indicates that complex equilibria occur in an intermediate regime on the NMR time scale. The foregoing evidence has been confirmed by the results obtained from molecular modeling studies. The 7-azaindole moiety of LOM1392 is positioned below the G17-G23 base pair of TP3-T6, leading to the formation of π–π stacking interactions (Figure 3a). The -CONH_2_ group is locked by an intramolecular hydrogen bond with the N_7_ of the 7-azaindole ring. The phenyl ring is located along the groove, interacting with the aromatic portion of G17 through π–π stacking interactions. The interaction pattern of LOM1392 to TP3-T6 is completed by two salt bridges between the quaternary nitrogen of piperidine and the OP_2_ residues of C18 and G22 (Figure 3a).

The same orientation was observed for MK4827. Again, the amide group is blocked by an intramolecular hydrogen bond with the indazole ring that lies below the G17-G23 base pair, allowing the formation of π–π stacking interactions. The phenyl group of MK4827 interacts with G17 via π–π stacking interactions, while the charged piperidine nitrogen forms a hydrogen bond with O3′G17 and two attractive charge interactions with the OP_2_ groups of C18 and G22 (Figure 4a).

**Figure 5:**
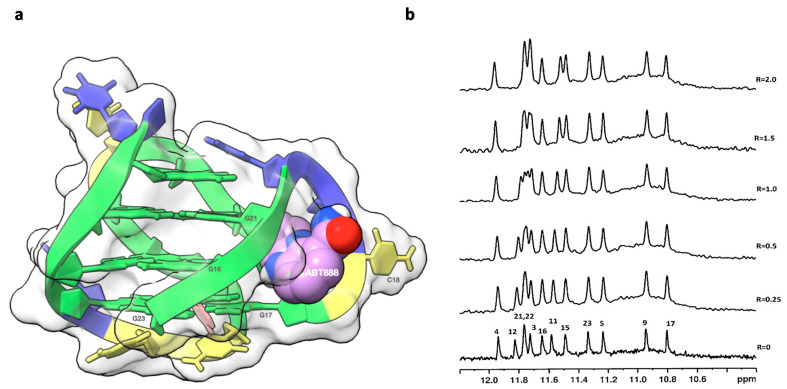
(**a**) ChimeraX [15] graphical representations of the complex and (**b**) imino proton region of the 1D NMR titration spectra of TP3-T6 with ABT888. The complex at TP3-T6 was obtained by molecular docking and optimized by molecular dynamics (MD), and it is represented as a side view of the ghostly-white solvent-accessible surface (SAS) of the TP3-T6 target. The nucleotides are rendered in stick and filled rings: cytosine in yellow, guanine in green, and thymine in blue. ABT888 is represented as van der Waals (vdW) spheres. The imino proton regions of the 1D NMR titration spectra were recorded at 25 °C in H_2_O/D_2_O (9:1), 20 mM of KH_2_PO_4_, and 70 mM of KCl pH = 7.0, at different R = [ABT888]/[DNA] ratios.

The same titration experiments were performed to test the binding process of ABT888 and AZD2281. Unlike the previously reported results, only very small changes in the imino proton signals were observed by adding ABT888 to the TP3-T6. The G residues of the 3′-end tetrad did not appear affected, as well as the residues of the middle tetrad, whereas the 5′-end residues tetrad was slightly shifted (Figure 5b).

**Figure 6:**
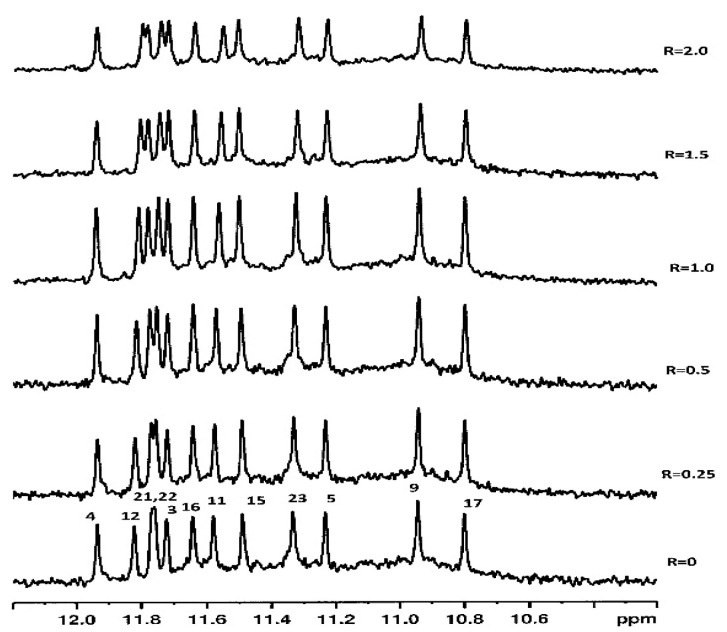
Imino proton region of the 1D NMR titration spectra of TP3-T6 AZD2281 at 25 °C in H_2_O/D_2_O (9:1), 20 mM of KH_2_PO_4_, and 70 mM of KCl pH = 7.0, at different R = [AZD2281/[DNA] ratios.

AZD2281 seemed to not give any perturbation at both 3′ and 5′end tetrads (Figure 6); thus, it was not selected for further investigation.

Again, the molecular modeling results are in agreement with the NMR findings. In fact, ABT888 is positioned completely within the groove. The benzimidazole ring is sandwiched between the OP_2_ groups of C18 and G22, held in place by π–anion interactions (Figure 5a). The amide group interacts with OP_2_T19 and OP_1_G21, giving rise to two hydrogen bonds. Finally, the quaternary nitrogen of ABT888 is held in place by three hydrogen bonds (with O3′G16, O5′G17, and O3′C18) and three salt bridges (with OP2G17, OP2T19, and OP2G21).

**Figure 7:**
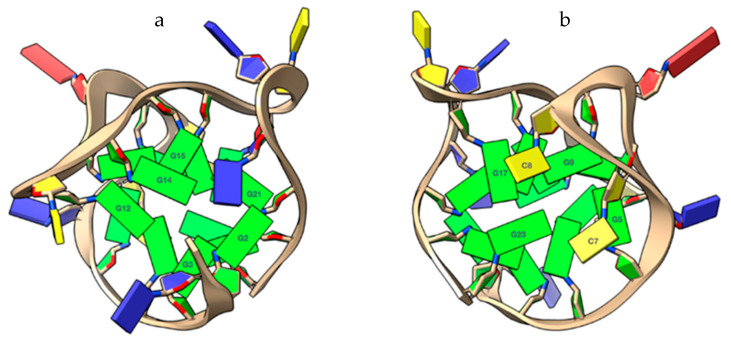
Close-up views of the capping at the top (**a**) and lower (**b**) ends of the TP3-T6 target, rendered with the software ChimeraX [15]. (**a**) The G2 and G14 bases at the 5′-end, interacting via reverse Hoogsteen bonding, prevent the ligands interacting with the 5′-end tetrad (G3-G12-G15-G21). (**b**) C7 and C8 bases, allowing the ligands a partial but effective π–π stacking interaction to the G17 and G23 bases at the 3′-end. Nucleotide bases are represented as slabs and filled sugars. Guanine residues are colored green, thymine residues are colored in blue, and cytosine residues are colored in yellow.

Overall, the results of molecular modeling studies clearly show the impossibility for the ligands to give effective π–π stacking interactions with the tetrad at the 5′-end. The steric hindrance deriving from the presence of G2 and G14 prevents ligands interacting effectively with the underlying G3-G12-G15-G21 tetrad (Figure 7a). This effect is also observed on the G5-G9-G17-G23 3′-end tetrad. However, in this case, the bases C7 and C8 only partially prevent the π–π stacking interactions with the tetrad at the 3‘-end (Figure 7b). Although G5 and G9 are not easily accessible to the ligands, their terminal aromatic moiety can still interact efficiently with G17 and G23, through classic π–π stacking interactions.

### 2.2. Fluorescence and CD Studies on the LOM1392, ABT888, and MK4827 Complexes with TP3-T6 (5′-D TGGGGT6CCGAGGCGGGGCTTGGG-3′)

**Figure 8:**
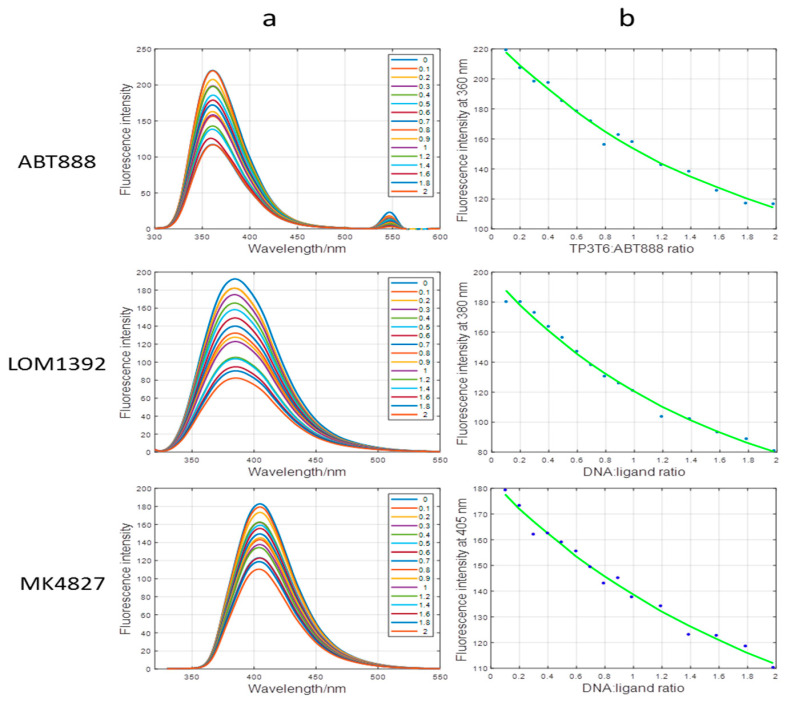
Fluorescence spectra (**a**) and fluorescence intensities measured at different wavelengths vs. DNA:ligand ratio (**b**) for ABT888, LOM1392, and MK4827. Experimental conditions are as explained in the text.

The interaction of TP3-T6 with the binders *LOM1392, ABT888*, and *MK4827* was analyzed by molecular fluorescence in order to calculate the binding constants by means of mole-ratio experiments. The compounds showed fluorescence in the buffer solution without TP3-T6. The addition of the oligonucleotide induced a reduction in fluorescence signal intensity of the binders. Additionally, the inverse titration of TP3-T6 with the binders was performed. In this case, the opposite effect was observed with an increasing fluorescence signal with the addition of the ligands. An estimation of the stoichiometry and the binding constants (Kb) relative to the interaction of these ligands with TP3-T6 was calculated by the EQUISPEC program. This program is based on multivariate analysis of the whole fluorescence spectra obtained during the titration [16]. For all compounds, the data analysis from the titration curves showed that the Kb were very similar for ABT888 and LOM1392 (5.48 ± 0.16 × 10^5^ M^−1^ and 5.45 ± 0.15 × 10^5^ M^−1^, respectively). MK4827 showed a Kb of 5.24 ± 0.20 × 10^5^ M^−1^. These values indicate a mild interaction in the formation of a 1:1 complex (Figure 8a,b).

**Figure 9:**
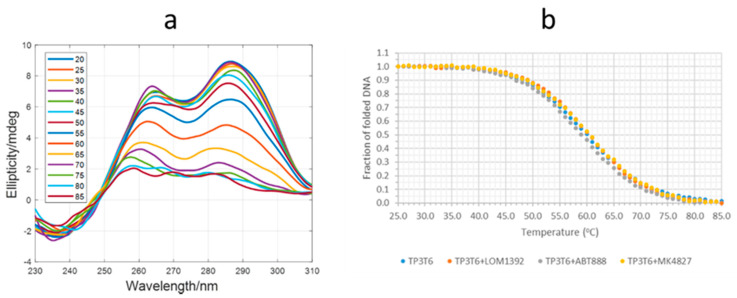
(**a**) CD spectra recorded along the melting of the TP3-T6:LOM1392 1:3 mixture. (**b**) CD melting curves (fraction of folded DNA) for TP3-TP6 and three mixtures. In all cases, the DNA and ligand concentrations were 2 and 6 μM, respectively, in 20 mM of phosphate buffer (pH 7.1) and 70 mM of KCl.

CD-monitored melting experiments were used to investigate the potential stabilization of the G-quadruplex structure adopted by TP3-T6 in the presence of the considered ligands. Figure 9a shows the CD spectra recorded along the melting of TP3-T6 in the presence of LOM1392 (1:3 mixture). See Supplementary Material for CD spectra recorded during the melting experiments of TP3-T6, and of TP3-T6:ABT888 and TP3-T6:MK4827 1:3 mixtures (Figure S1). The CD spectrum of the TP3-T6:LOM1392 mixture at 20 °C shows positive signals at 265 and 285 nm, which could be related to a hybrid parallel/antiparallel structure. With the increase in temperature, the CD bands disappear due to the unfolding of the structure. From the CD traces at 285 nm, the fraction of folded DNA was calculated assuming a two-state process. This assumption was checked by means of multivariate analysis of the whole dataset shown in Figure 9 [17].

These curves (Figure 9b) show that the addition of LOM1392, ABT888, and MK4827 produced little changes to the stability of the TP3-T6 G-quadruplex structure. From the CD melting curves, thermodynamic parameters associated with the unfolding were calculated (Table 1). Very little changes were observed in the presence of the ligand, in agreement with the relatively low values of the binding constants determined previously.

## 3. Materials and Methods

### 3.1. Ligands

Compound ABT888 was purchased from ChemScene LLC, Monmouth Junction, NJ, USA. The corresponding hydrochloride was prepared by treatment with 4 M of HCl in dioxane.

Compound MK4827 was purchased from Key Organics, Cornwall, UK. The corresponding hydrochloride was prepared by treatment with 4 M of HCl in dioxane.

Compound AZD2281 (Olaparib) was purchased from Selleckchem, Houston, TX, USA.

Compound LOM1392 was prepared as reported in the literature [9]. The corresponding hydrochloride was prepared by treatment with 4 M of HCl in dioxane.

### 3.2. Sample Preparation

The oligonucleotide was synthesized in 1 μmol scale on an Applied Biosystems DNA/RNA 3400 synthesizer, Foster City, USA by solid-phase 2-cyanoethylphosphoroamidite chemistry. It was passed through a Dowex 50WX2, cation exchange resins, and then desalted with a Sephadex (NAP-10) G25 column. The NMR samples of TP3-T6,5′ dTGGGGTCCGAGGCGGGGCTTGGG-3′ were prepared at 0.24–0.48 mM in the G-quadruplex concentration range, in H_2_O/D_2_O (9:1) containing 20 mM of KH_2_PO_4_ and 70 mM of KCl pH = 7.0. The samples were heated to 85 °C for 1 min and then cooled at room temperature overnight. A stock solution of drugs was prepared in DMSO-*d*_6_ at the concentration range of 16–17 mM. As the presence of DMSO at high concentrations may alter the structure of DNA, the amount of DMSO added to the sample was carefully monitored. The total amount of DMSO present in the final solution of the complexes was <7%, as this concentration did not affect the G-quadruplex structure.

### 3.3. NMR Experiments

The NMR spectra were recorded on a Bruker AV600 spectrometer, Hanau, Germany, operating at a frequency of 600.10 MHz for the ^1^H nucleus at 25 °C. ^1^H NMR titrations were performed by adding increasing amounts of the drug to the oligonucleotide solution until R = [ligand]/[DNA] = 2.0.

The G-quadruplex TP3-T6 5′-d(TGGGGTCCGAGGCGGGGCTTGGG)-3′ signals were previously assigned [13]. Phase-sensitive NOESY spectra were acquired at an R ratio R = [ligand]/[DNA] = 2.0 at 25 °C. A TPPI mode was used with 2048 × 1024 complex FIDs. Mixing times ranged from 200 ms to 400 ms. All spectra were transformed and weighted with a 90°-shifted sine-bell squared function to 4 K × 4 K real data points.

### 3.4. CD and Fluorescence

CD spectra were recorded on a Jasco J-810 spectropolarimeter, Grob-Umstadt, Germany, equipped with a Peltier temperature control unit (Seelbach, Germany). The DNA solution of TP3-T6 was transferred to a covered cell and the ellipticity was recorded with a heating rate of approximately 0.4 °C·min^−1^. Simultaneously, CD spectra were recorded every 5 °C from 220 to 310 nm. The spectrum of the buffer was subtracted. Each sample was allowed to equilibrate at the initial temperature for 30 min before the melting experiment began. In all experiments, the concentration of DNA was kept constant (2 µM), whereas the concentration of the considered ligands was increased. The medium consisted of 20 mM of phosphate buffer (pH 7.1) and 70 mM of KCl [13].

Molecular fluorescence spectra were measured with a JASCO FP-6200 spectrofluorimeter, Tokyo, Japan. The temperature was controlled at 20 °C using a water bath. The fluorescence spectra were monitored using a quartz cuvette with a 10 mm path length, with the excitation and emission slits set at 10 nm, and the scan speed at 250 nm/min. Measurements were taken at 271, 254, and 307 nm excitation wavelengths for ABT888, LOM1392, and MK4827, respectively. The buffer consisted of 20 mM of phosphate buffer (pH 7.1) and 70 mM of KCl. In all experiments, the concentration of the ligands was 3 µM, whereas the concentration of TP3-T6 at the 69 µM sequence was increased.

The determination of the ratio ligand:DNA and the calculation of the binding constants was performed from the fluorescence data recorded along titrations of ligands with DNAs by using the EQUISPEC program [16]. This program is based on the multivariate analysis of the whole spectra measured along the titration.

### 3.5. Molecular Modeling Studies

The starting TP3-T6 3D-structure was taken from the first model of the NMR ensemble deposited in Protein Data Bank (PDB accession code: 6AC7 [13]).

Flexible docking calculations for each ligand at the Tp3-T6 target were performed by AutoDock 4.2, The Scripps Research Institute, San Diego, USA [18,19]. The Lamarckian Genetic Algorithm in combination with a grid-based energy evaluation method was employed to calculate grid maps, in an 80 Å × 80 Å × 80 Å box centered on the macromolecule center of mass (COM) and with a spacing of 0.01 Å. Gasteiger–Marsili charges [20] were added to the ligand by using AutoDock Toolkit (ADT) [21], and the phosphorus atoms in the DNA were parameterized using the Cornell parameters. The solvation parameters were added to the system by means of the Addsol utility of AutoDock. For each ligand, the initial population consisted of 100 randomly placed individuals, with a maximum number of 250 energy evaluations and an elitism value of 1, a mutation rate of 0.02, and a crossover rate of 0.80. The local search for the ligand was conducted using 250 independent docking runs by applying the so-called pseudo-Solis and Wets algorithm with a maximum of 250 iterations per local search. The docking results were scored by using an in-house version of the simpler intermolecular energy function based on the Weiner force field, and the lowest energy conformations (differing by less than 1.0 Å in positional root-mean-square deviation (rmsd)) were collected.

The complexes resulting from the molecular docking step were placed at the center of a box with boundaries at 2.0 nm apart from all atoms and solvated with TIP3P water molecules. The amber ff99 force field [22] with bsc1 corrections [23] was used to describe the TP3-T6 G-Quadruples. Then, 1000 steps of minimization were performed on the initial systems to remove bad contacts, followed by a heating ramp of short (100 ps) consecutive simulations at 50, 100, 150, 200, 250, and 298 K. The production simulations consisted of 5 ns of Langevin [24,25] molecular dynamics (MD) NPT equilibration at 298 K and 1 atm, as implemented in NAMD [26]. During equilibration, water molecules were unrestrained and periodic boundary conditions were applied in all spatial dimensions. All bonds to hydrogen atoms were constrained using the SHAKE [27] algorithm. The water molecules were kept rigid with SETTLE [28], allowing an integration time step of 0.002 ps. A Berendsen thermostat (coupling time of 0.1ps) was applied to the systems [29], and the electrostatic interactions were calculated using the Particle Mesh Ewald (PME) [30,31] method (Coulomb cut-off radius of 1.2 nm).

Molecular graphics and analyses were performed with UCSF ChimeraX, developed by the Resource for Biocomputing, Visualization, and Informatics at the University of California, San Francisco, with support from the National Institutes of Health R01-GM129325 and the Office of Cyber Infrastructure and Computational Biology, National Institute of Allergy and Infectious Diseases [15].

The schematic block representations in Figure 2 were created with DSSR (Dissecting the Spatial Structure of RNA), an integrated computational tool for the analysis and annotation of 3D nucleic acid structures [32].

## 4. Conclusions

PARP1 emerged as an attractive target for cancer therapy as it is involved in DNA repair processes. Several PARP1 inhibitors have been recently developed and approved for clinical treatments. Due to the rapid insurgence of resistance, alternative strategies are required to selectively regulate PARP1 activity. Recent experiments have identified a noncanonical G-quadruplex-forming sequence containing bulges within the PARP1 promoter. This important finding opens a new avenue of investigation. We were particularly intrigued by the possibility of identifying compounds acting as PARP inhibitors by a dual approach. In particular, we explored the ability of PARP1 enzyme inhibitors to simultaneously regulate PARP1 expression via G-quadruplex DNA targeting, by an integrated study of NMR, CD, fluorescence, and molecular modeling.

Considering that no PARP promoter modulator has been identified so far, we started our investigation by studying clinically used PARP1 inhibitors such as veliparip (ABT 888), olaparib (AZD 2281), and niraparib (MK4827). We also selected compound LOM 1392, which belongs to a new class of PARP1 inhibitors with a 7-azaindole-1-carboxamide core and is a strong *c-myc* and telomere G-quadruplex binder. From our experiments, it emerged that two out of the four compounds showed a mild interaction with the G quadruplex target, whereas the remaining two did not cause any perturbation of the structure. Molecular modeling studies confirmed the experimental results. Compound ABT 888, missing the central aromatic core present in MK4827, is not able to stably interact with the G quadruplex-forming structures. Indeed, it inserts in a groove without any perturbation of the tetrads. On the contrary, AZD2281, with a methylene spacer connecting the central aromatic core to the heterocyclic ring and lacking the protonable amino group found in LOM1392 and MK4827, does not interact with the oligonucleotides. The results confirm that the structural requirements for the interaction are quite strict. Indeed, in a previous work [14], we reported that LOM 1392 strongly binds the G-quadruplexes of a model of human telomere sequence (d(TTA GGG T)4), and a model of the c-MYC promoter Pu22 sequence. In the first case, LOM 1392 was located between A3 and G4 units and over the G6 residue, while it formed a 2:1 complex with a Pu22 quadruplex, with the two molecules located over the external tetrads at the 5′ and 3′-ends. This different behavior clearly confirms the unique structural features of TP3 and makes the search of specific ligands very challenging.

The studied compounds, despite their low affinity with the target, can be considered as a starting point toward the identification of strongly interacting ligands obtained by structural modifications of the scaffolds. Rational modification of the most promising scaffolds, also guided by molecular modeling support, could allow the obtaining of selective PARP G-quadruplex modulators, endowed at the same time with activity on the enzyme.

## Figures and Tables

**Table 1:** Thermodynamic parameters associated with the folding of the G-quadruplex structure in the absence and presence of several ligands. A two-state process was assumed.

System	T_m_ (°C)	ΔH (kcal·mol^−1^)	ΔS (cal·K^−1^·mol^−1^)	ΔG_37 °C_ (kcal·mol^−1^)
TP3T6	59.6 ± 0.8	−39.0 ± 0.4	−117.2 ± 1.0	−2.7 ± 1.1
TP3T6:LOM1392	60.2 ± 1.0	−43.1 ± 0.5	−129.2 ± 1.5	−3.0 ± 1.6
TP3T6:ABT888	58.8 ± 1.3	−41.1 ± 0.6	−123.7 ± 1.9	−2.7 ± 2.0
TP3T6:MK4827	60.3 ± 1.3	−41.1 ± 0.6	−123.0 ± 1.8	−3.0 ± 1.9

## Data Availability

Not applicable.

## Supplementary Materials

The following are available online at https://www.mdpi.com/article/10.3390/ijms22168737/s1.
