# Supplementary material for: Investigation of the Complexes Formed between PARP1 Inhibitors and PARP1 G-Quadruplex at the Gene Promoter Region
Source: Int J Mol Sci. 2021 Aug 14;22(16):8737. doi: 10.3390/ijms22168737 (PMC8395737; doi:10.3390/ijms22168737)
Supplement: Supplementary file 1 [file ijms-22-08737-s001.zip › ijms-1297195-supplementary.pdf]

Investigation of the Complexes Formed between PARP1 Inhibitors and PARP1 G-Quadruplex at the Gene Promoter Region

Sabrina Dallavalle [1,2], Salvatore Princiotto [1], Luce M. Mattio [1], Roberto Artali [3], Loana Musso [1], Anna Aviñó [4], Ramon Eritja [4], Claudio Pisano [5], Raimundo Gargallo [5] and Stefania Mazzini [1,*]

[1] Department of Food, Environmental and Nutritional Sciences (DEFENS), University of Milan (Università degli Studi di Milano), 20133 Milan, Italy; sabrina.dallavalle@unimi.it (S.D.); salvatore.princiotto@unimi.it (S.P.); luce.mattio@unimi.it (L.M.M.); loana.musso@unimi.it (L.M.)
[2] National Institute of Fundamental Studies, Kandy 20000, Sri Lanka
[3] Scientia Advice di Roberto Artali, 20832 Desio, Italy; roberto.artali@scientia-advice.com
[4] Institute for Advanced Chemistry of Catalonia (IQAC), CSIC, Networking Center on Bioengineering, Biomaterials and Nanomedicine (CIBER-BBN), 08034 Barcelona, Spain; aaagma@cid.csic.es (A.A.); recgma@cid.csic.es (R.E.)
[5] Biogem, Research Institute, Ariano Irpino, 83100 Avellino, Italy; claudio.pisano@biogem.it (C.P.); raimon_gargallo@ub.edu (R.G.)
[6] Department of Chemical Engineering and Analytical Chemistry, University of Barcelona, 08028 Barcelona, Spain
* Correspondence: stefania.mazzini@unimi.it

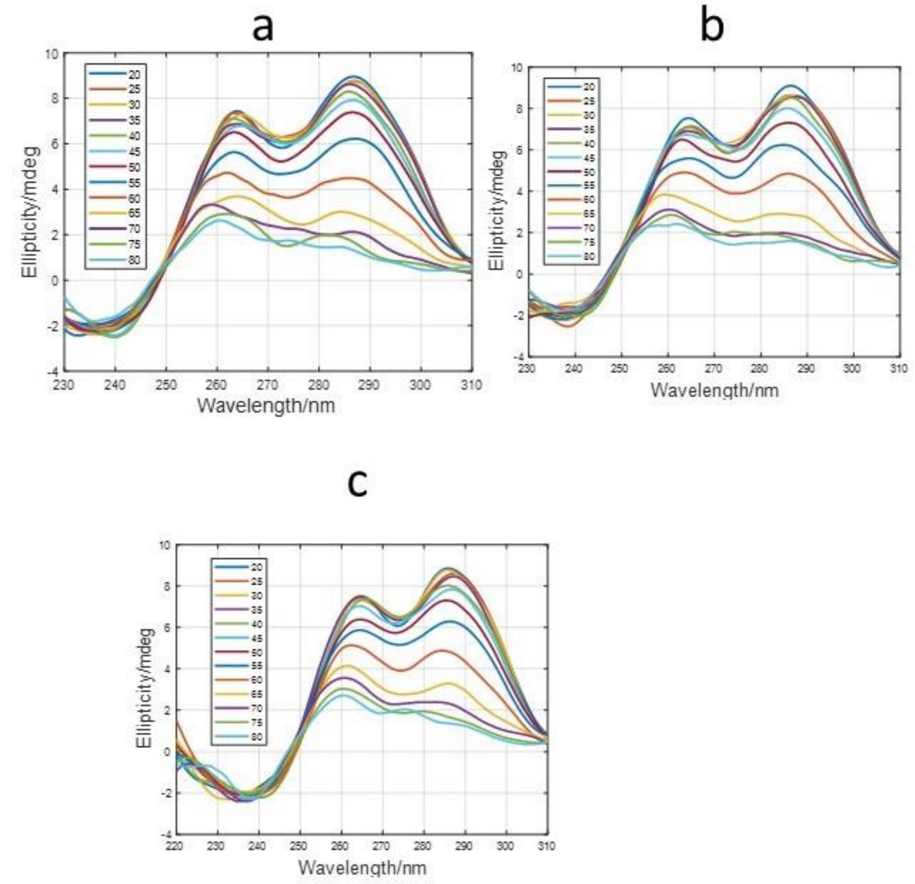

Figure S1. CD spectra recorded along the melting experiments of (a) TP3-T6, (b) TP3-T6:ABT888, and (c) TP3-T6:MK4827. In all cases, DNA and ligand concentration were 2 and 6 mM, respectively, 20 mM phosphate buffer (pH 7.1), 70 mM KCl.
